# Bone Marrow-Derived Mesenchymal Stem Cells Migrate toward Hormone-Insensitive Prostate Tumor Cells Expressing TGF-β via N-Cadherin

**DOI:** 10.3390/biomedicines9111572

**Published:** 2021-10-29

**Authors:** Jinok Noh, Jinyeong Yu, Wootak Kim, Aran Park, Ki-Sook Park

**Affiliations:** 1 Department of Biomedical Science and Technology, Graduate School, Kyung Hee University, Seoul 02447, Korea; rjo8853@khu.ac.kr (J.N.); kimwotak@khu.ac.kr (W.K.); 2 Graduate School of Biotechnology, Kyung Hee University, Yongin 17104, Korea; jinyeong90@khu.ac.kr (J.Y.); arvi2114@khu.ac.kr (A.P.); 3 East-West Medical Research Institute, Kyung Hee University, Seoul 02447, Korea

**Keywords:** bone marrow-derived mesenchymal stem cell, prostate tumor, tumor microenvironment, N-cadherin, TGF-β

## Abstract

The prostate tumor microenvironment plays important roles in the metastasis and hormone-insensitive re-growth of tumor cells. Bone marrow-derived mesenchymal stem cells (BM-MSCs) are recruited into prostate tumors to facilitate tumor microenvironment formation. However, the specific intrinsic molecules mediating BM-MSCs’ migration to prostate tumors are unknown. BM-MSCs’ migration toward a conditioned medium (CM) of hormone-insensitive (PC3 and DU145) or hormone-sensitive (LNCaP) prostate tumor cells was investigated using a three-dimensional cell migration assay and a transwell migration assay. PC3 and DU145 expressed transforming growth factor-β (TGF-β), but LNCaP did not. Regardless of TGF-β expression, BM-MSCs migrated toward the CM of PC3, DU145, or LNCaP. The CM of PC3 or DU145 expressing TGF-β increased the phosphorylation of Smad2/3 in BM-MSCs. Inactivation of TGF-β signaling in BM-MSCs using TGF-β type 1 receptor (TGFBR1) inhibitors, SB505124, or SB431542 did not allow BM-MSCs to migrate toward the CM. The CM of PC3 or DU145 enhanced N-cadherin expression on BM-MSCs, but the LNCaP CM did not. SB505124, SB431542, and TGFBR1 knockdown prevented an increase in N-cadherin expression. N-cadherin knockdown inhibited the collective migration of BM-MSCs toward the PC3 CM. We identified N-cadherin as a mediator of BM-MSCs’ migration toward hormone-insensitive prostate tumor cells expressing TGF-β and introduced a novel strategy for controlling and re-engineering the prostate tumor microenvironment.

## 1. Introduction

Prostate tumors are the most frequent malignancy and the second leading cause of cancer-related death in men worldwide [1]. Androgen deprivation therapy is the primary treatment for metastatic prostate tumors through medical or surgical castration. However, patients frequently develop castration resistance, making prostate tumor cells resistant to androgen deprivation therapy despite the retention or amplification of the androgen receptor in prostate tumor cells [1,2,3]. The tumor microenvironment of prostate tumors consists of various non-tumor cells, including immune cells, endothelial cells, and fibroblasts [1,4]. The functional interaction between tumor microenvironmental cells and tumor cells plays important roles in prostate tumor progress, the resistance of tumor cells to chemotherapy, and the change of androgen-sensitive prostate tumor cells into androgen-insensitive cells [3,4,5,6].

Mesenchymal stem cells of bone marrow are rare and multipotent stem cells that can be differentiated into different cell types of skeletal lineage, such as osteoblasts [7]. They play essential roles in the maintenance of bone marrow homeostasis and in the formation of a microenvironment for hematopoietic stem cells inside of bone marrow [8,9,10]. Bone marrow-derived mesenchymal stem cells (BM-MSCs) can be recruited into injured tissues to participate in the repair and regeneration of the injury [11]. Injured tissues secrete various factors, including transforming growth factor-β (TGF-β), to recruit BM-MSCs [12,13,14]. BM-MSCs have also been shown to be recruited into various types of solid tumors and transdifferentiated into cancer-associated fibroblasts to contribute to the formation of a tumor-tropic microenvironment [15,16,17,18]. Prostate tumors recruit BM-MSCs using various factors such as CXC chemokine ligand (CXCL) 16, CCL5, and TGF-β [17,18,19]. The recruited BM-MSCs promote an epithelial–mesenchymal transition of prostate tumor cells, which eventually enhances metastasis [17]. BM-MSCs that have migrated to prostate tumors also promote the resistance of tumor cells to chemotherapy such as docetaxel [19,20], as well as resistance to androgen deprivation therapy [21]. Although it has been demonstrated that the migration of BM-MSCs to prostate tumors plays essential roles in tumor progression, the cellular and molecular mechanisms of BM-MSC migration to prostate tumors have not been elucidated yet.

BM-MSCs are recruited into prostate tumors in response to TGF-β expressed by prostate tumors [18]. The expression level of TGF-β in prostate tumors has a positive correlation with the aggressive features of prostate tumors or the Gleason score [22,23]. A strong correlation between the elevated plasma level of TGF-β and prostate tumor progression has also been found [24,25]. TGF-β functions as a tumor suppressor in normal prostate epithelial cells and early prostate tumor cells; it inhibits their proliferation and induces apoptosis. However, it functions as a tumor promoter in metastatic prostate tumors [26,27,28]. TGF-β signaling regulates the dependence of prostate tumor cells on androgen and the acquisition of androgen insensitivity [28,29]. TGF-β signaling is transduced by heterodimeric receptor complexes composed of type 1 and type 2 receptors [30]. The type 2 receptor phosphorylates the type 1 receptor in response to the binding of the TGF-β ligand and the phosphorylated type 1 receptor, then phosphorylates Smad2/3, which regulates the transcription of TGF-β downstream genes via the formation of a complex with Smad4 [30,31].

Our previous studies demonstrated that the expression of neural cadherin (N-cadherin) increases upon cell–cell contact between BM-MSCs, and that BM-MSCs migrate via N-cadherin in response to TGF-β and breast tumor cells expressing TGF-β [32,33]. Cadherins are transmembrane proteins that mediate cell–cell adhesion via Ca2+-dependent homophilic interactions in their extracellular domain [34]. Cadherins include N-cadherin and osteoblast-cadherin, both of which BM-MSCs express [32,35]. Cadherins form adherens junctions. The cytoplasmic domain of cadherins interacts directly with p120 and β-catenin, and indirectly with α-catenin, which is associated with actin [34]. N-cadherin forms adherens junction-like structures upon cell–cell contact between BM-MSCs [32]. Moreover, N-cadherin-mediated adhesion of BM-MSCs is required for their collective migration toward breast tumor cells [32]. In the present study, we investigated the role of N-cadherin expressed on BM-MSCs in terms of their motility toward prostate tumor cells.

## 2. Materials and Methods

### 2.1. Cell Lines and Cell Culture

PC3 cells were obtained from the American Type Culture Collection (ATCC; Manassas, VA, USA). DU145 and LNCaP cells were obtained from the Korean Cell Line Bank (Seoul, South Korea). PC3 and DU145 cells were cultured in Dulbecco’s Modified Eagle’s medium/high glucose (DMEM/high, Cytiva, Marlborough, MA, US), supplemented with 10% heat-inactivated fetal bovine serum (FBS; Invitrogen, Carlsbad, CA, USA), 1% l-glutamine (Invitrogen), and 1% penicillin and streptomycin (P/S; Invitrogen). LNCaP cells were cultured in Roswell Park Memorial Institute 1640 (RPMI 1640, Invitrogen) supplemented with 10% FBS and 1% P/S. Human bone marrow-derived mesenchymal stem cells (BM-MSCs; Lonza, Basel, Switzerland) were cultured in Mesenchymal Stem Cell Growth Medium (Lonza). For the subculture of BM-MSCs, they were detached with TrypLE (Invitrogen). For all experiments, BM-MSCs between passages 5 and 7 were used. DMEM (Cytiva) supplemented with 10% FBS, 1% l-glutamine, and 1% P/S was used for migration experiments.

### 2.2. Conditioned Medium

PC3, DU145, or LNCaP cells were cultured with their normal culture media. After culturing for confluence, the cells were washed in phosphate-buffered saline (PBS). Then, the cells were cultured with DMEM/high supplemented with 1% l-glutamine and 1% P/S for 2 days. To prepare the conditioned medium (CM) for the control, DMEM/high supplemented with 1% l-glutamine and 1% P/S was incubated for 2 days under cell-free conditions. Conditioned medium from PC3 (PC3 CM), DU145 (DU145 CM), LNCaP (LNCaP CM), or the control (CON CM) was filtered through a 0.2 µm filter (CORNING, Corning, NY, USA), aliquoted, and stored at −80 °C before use.

### 2.3. Transwell Migration Assay

BM-MSCs were incubated using DMEM supplemented with 2% FBS, 1% l-glutamine, and 1% P/S for 18 h. The BM-MSCs were seeded at a density of 3 × 10^4^ cells/cm^2^ and cultured for 6 h in the upper chamber of a Millicell culture plate insert (8 μm pore size; EMD Millipore, Burlington, MA, USA) coated with Type 1P collagen (5 μg/mL; Nitta Gelatin NA Inc., Osaka, Japan). Thereafter, the media in the upper chamber of each insert were replaced with serum-free DMEM including 1% l-glutamine and 1% P/S. PC3 CM, DU145 CM, LNCaP CM, or CON CM was added to the lower chamber of each insert. If necessary, the BM-MSCs were pretreated with SB431542 (10 μM; Tocris, Bristol, UK) or SB505124 (5 μM; Sigma, St. Louis, MO, USA) for 30 min prior to treatment with CM. The cells were then incubated for 12 h, fixed with 4% paraformaldehyde (PFA, Sigma) in PBS for 2 h at 25 °C, washed with PBS, and then stained using 4,6-diamidino-2-phenylindole (DAPI, Invitrogen) for 10 min. The membranes were mounted with Fluoromount-G solution (SouthernBiotech, Birmingham, AL, USA). Cells on either the lower or upper surface of five randomly selected areas of each Millicell membrane were imaged using a Zeiss LSM 700 confocal microscope (Carl Zeiss, Oberkochen, Germany). The number of cells in each image was counted using Adobe Photoshop CS6 version 13.0.1 (Adobe Systems Incorporated, San Jose, CA, USA), and the number of migrated cells was determined as a percentage of total cells on both sides of the insert.

### 2.4. Three-Dimensional (3D) Cell Migration Assay

A BM-MSC–collagen mixture was prepared at a density of 5 × 10^5^ cells/100 μL using a Type 1A collagen solution (final concentration, 1.5 mg/mL; Nitta Gelatin Inc.), reconstitution buffer (2.2 g NaHCO_3_ and 200 mM HEPES in 0.05 N NaOH), 10× Minimum Essential Medium (Invitrogen), and sterile ultrapure distilled water. Three microliters of the cell–collagen mixture was solidified in a μ-dish 35 mm dish, low Grid-500 (ibidi, Munich, Germany), and incubated for 24 h with PC3 CM, DU145 CM, LNCaP CM, or CON CM after starvation using serum-free DMEM for 6 h. Images were captured using a Nikon ECLIPSE TS 100 inverted microscope (Nikon Instruments Inc., Melville, NY, USA), and the number of all migrating cells from the cell–collagen mixture was counted using Adobe Photoshop CS6 (Adobe Systems Incorporated, San Jose, CA, USA). The effects of N-cadherin on migrating BM-MSCs was evaluated using a cell–collagen mixture containing BM-MSCs transfected with N-cadherin siRNA and BM-MSCs transfected with the control siRNA. BM-MSCs transfected with the control siRNA (2.5 × 10^5^ cells) were mixed with BM-MSCs transfected with N-cadherin siRNA (2.5 × 10^5^ cells) in 100 μL of the Type 1A collagen solution. The control siRNA transfected-BM-MSCs were stained with calcein AM (Cayman Chemical, Ann Arbor, MI, USA) before mixing. Migrating cells on the surface of 13 randomly selected areas were imaged using a Zeiss LSM 700 confocal microscope (Carl Zeiss) to analyze the number of migrating BM-MSCs.

### 2.5. Western Blot Analysis

BM-MSCs were washed twice with ice-cold PBS. They were then incubated with 2× SDS buffer (100 mM Tris-HCl (pH 6.8), 2% (*w/v*) SDS, 0.01% bromophenol blue, 20% glycerol, and 1.43 M β-mercaptoethanol) for 5 min at 25 °C. Then, the proteins were denatured at 95 °C for 5 min. Western blot analysis was performed with the following primary antibodies: Anti-phospho-Smad 2/3 (1:1000; Cell Signaling Technology, Danvers, MA, USA), anti-Smad2 (1:1000; Cell Signaling Technology), anti-N-cadherin (1:2000; Invitrogen), and anti-α-tubulin (1:20,000; Sigma). Densitometry of the bands obtained was carried out with ImageJ software (NIH, Bethesda, MD, USA).

### 2.6. Real-Time Polymerase Chain Reaction (RT-PCR)

Total RNA was extracted using the TRIzol reagent (Invitrogen), and cDNA was synthesized with SuperScript III Reverse Transcriptase (Invitrogen), according to the manufacturer’s protocol. Quantitative real-time reverse transcription–polymerase chain reaction (qRT-PCR) was performed using Power SYBR Green PCR Master Mix (Invitrogen). Ribosomal protein S9 gene (RPS9) was used as an endogenous control. The primers used to detect the expression levels of RPS9, N-cadherin, and TGFBR1 were as follows: RPS9 (sense): 5′-CTGACGCTTGATGAGAAGGAC-3′, RPS9 (antisense): 5′-CAGCTTCATCTTGCCCTCAT-3′; N-cadherin (sense): 5′-CATCCCTCCAATCAACTTGC-3′, N-cadherin (antisense): 5′-ATGTGCCCTCAAATGAAACC-3′; TGFBR1 (sense): 5′-ATTACCAACTGCCTTATTATGA-3′, TGFBR1 (antisense): 5′-CATTACTCTCAAGGCTTCAC-3′. The expression of TGF-β1, TGF-β2, and TGF-β3 was analyzed by semi-quantitative RT-PCR using i-Taq DNA Polymerase (iNtRON Biotechnology, Gyeonggi-do, Seongnam, South Korea) and qRT-PCR. The glyceraldehyde 3-phosphate dehydrogenase gene (GAPDH) was used as an endogenous control for semi-quantitative RT-PCR analysis. GAPDH, TGF-β1, TGF-β2, and TGF-β3 were as follows: GAPDH (sense): 5′- CAGCCTCAAGATCATCAGCA-3′, GAPDH (antisense): 5′-TGTGGTCATGAGTCCTTCCA-3′; TGF-β1 (sense): 5′-CAACACATCAGAGCTCCGAGAA-3′, TGF-β1 (antisense): 5′-AAGGCGAAAGCCCTCAATTT-3′; TGF-β2 (sense): 5′-ATGCGGCCTATTGCTTTAGA-3′, TGF-β2 (antisense): 5′-TAAGCTCAGGACCCTGCTGT-3′; TGF-β3 (sense): 5′-ATGACCCACGTCCCCTATCA-3′, TGF-β3 (antisense): 5′-CAGACAGCCAGTTCGTTGTG-3′.

### 2.7. Transfection of Cells with Small Interfering RNAs (siRNAs)

BM-MSCs were seeded in 6-well plates at a density of 1.3 × 10^5^ cells/well and incubated in the culture medium for 24 h. BM-MSCs were then transfected with the siRNAs using Lipofectamine RNAiMax reagent (Invitrogen). The siRNAs for the experiments were as follows: Scrambled negative control siRNAs (Origene, Rockville, MD, USA), N-cadherin siRNAs (Invitrogen), TGF-β type 1 receptor (TGFBR1) siRNAs #1 (sense: 5′-GAGAAAGUGGCCAUUUACAUU-3′; antisense: 5′-UGUAAAUGGCCACUUUCUCUU-3′; Genolution, Seoul, South Korea), TGFBR1 siRNAs #2 (sense: 5′-GGGUCUGUGACUACAACAUUU-3′; antisense: 5′-AUGUUGUAGUCACAGACCCUU-3′; Genolution). A mixture with equal amounts of TGFBR1 siRNAs #1 and #2 was used to knockdown TGFBR1.

### 2.8. Immunocytochemistry

BM-MSCs were seeded on cover slips coated with type 1P collagen (1 × 10^4^ cells/cm^2^) and cultured in the culture medium for 24 h. Then, the cells were starved with serum-free DMEM supplemented with 1% l-glutamine and 1% P/S for another 18 h. The cells were then treated with PC3 CM or CON CM for 24 h, and subsequently fixed with 4% PFA. After fixation, immunocytochemistry was performed according to standard protocols [32]. The following primary antibodies were used: Anti-N-cadherin (1:30; Invitrogen), anti-N-cadherin (1:100; Takara, Shiga, Japan), anti-β-catenin (1:100; Bethyl Laboratories, Montgomery, TX, USA), and anti-α-catenin (1:100; BD Biosciences, San Jose, CA, USA). Actin was stained with Alexa Fluor 633-tagged phalloidin (1:1000; Invitrogen). Nuclei were stained using DAPI for 10 min. Images were taken using a Zeiss LSM 700 confocal microscope (Carl Zeiss) at a 400× magnification. The number and the length of N-cadherin-positive structures at the borders between BM-MSCs and the width of N-cadherin-positive borders between BM-MSCs were measured (Figure S1) using Adobe Photoshop CS6 (Adobe Systems Incorporated, San Jose, CA, USA).

### 2.9. Statistical Analysis

Data are expressed as mean or mean ± SD. Statistical significance was analyzed using two-way ANOVAs and two-tailed Student’s *t*-tests. GraphPad version 6.07 (GraphPad Software Inc., San Diego, CA, USA) was used for statistical analysis, and *p*-values < 0.05 were considered significant.

## 3. Results

### 3.1. BM-MSCs Migrate in Response to Prostate Tumor Cells

**Figure 1:**
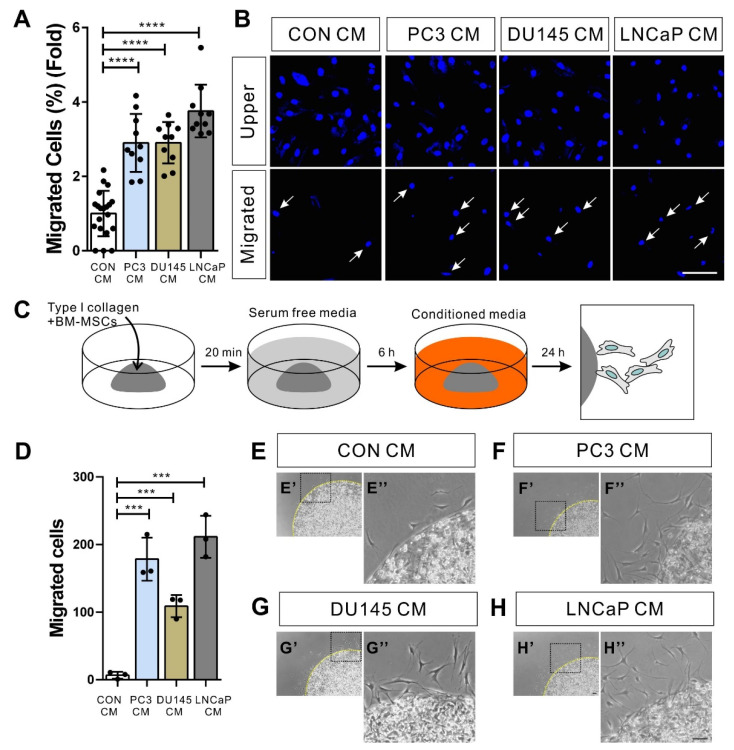
Bone marrow-derived mesenchymal stem cells (BM-MSCs) migrate toward the prostate tumor cell-conditioned medium. (**A**,**B**) Migration of BM-MSCs in response to the control-conditioned medium (CON CM), the PC3-conditioned medium (PC3 CM), the DU145-conditioned medium (DU145 CM) or the LNCaP-conditioned medium (LNCaP CM). Results are presented as the mean ± SD (*n* = 2 samples for each group, 4 independent experiments). White arrows indicate DAPI-stained nuclei of migrated BM-MSCs on the lower membrane surface. (**C**) Schematic representation of 3D migration assay. (**D**–**H**) Three-dimensional migration of BM-MSCs toward CON CM, PC3 CM, DU145 CM, or LNCaP CM. Results are presented as the mean ± SD (*n* = 3 samples for each group, 3 independent experiments). Black dotted boxes in E’, F’, G’, and H’ are magnified in E”, F”, G”, and H”, respectively. Yellow broken lines indicate the margin of collagen gel. Scale bars indicate 100 μm. *p*-Values were obtained by *t*-test (*** *p* ≤ 0.001; **** *p* ≤ 0.0001).

We first examined whether hormone-insensitive and -sensitive prostate tumor cells induce the migration of BM-MSCs. The human prostate tumor cell lines DU145 and PC3 are androgen-insensitive prostate tumor cells [36]. In contrast, LNCaP is a hormone-sensitive prostate tumor cell [37]. BM-MSCs migrated toward the conditioned medium of PC3 (PC3 CM), DU145 (DU145 CM), or LNCaP (LNCaP CM) (Figure 1A,B). The migration of BM-MSCs in response to the conditioned medium was confirmed using a 3D cell migration assay [32,38] (Figure 1C–H).

### 3.2. TGF-β Signal Mediates BM-MSCs’ Migration toward Hormone-Insensitive Prostate Tumor Cells

**Figure 2:**
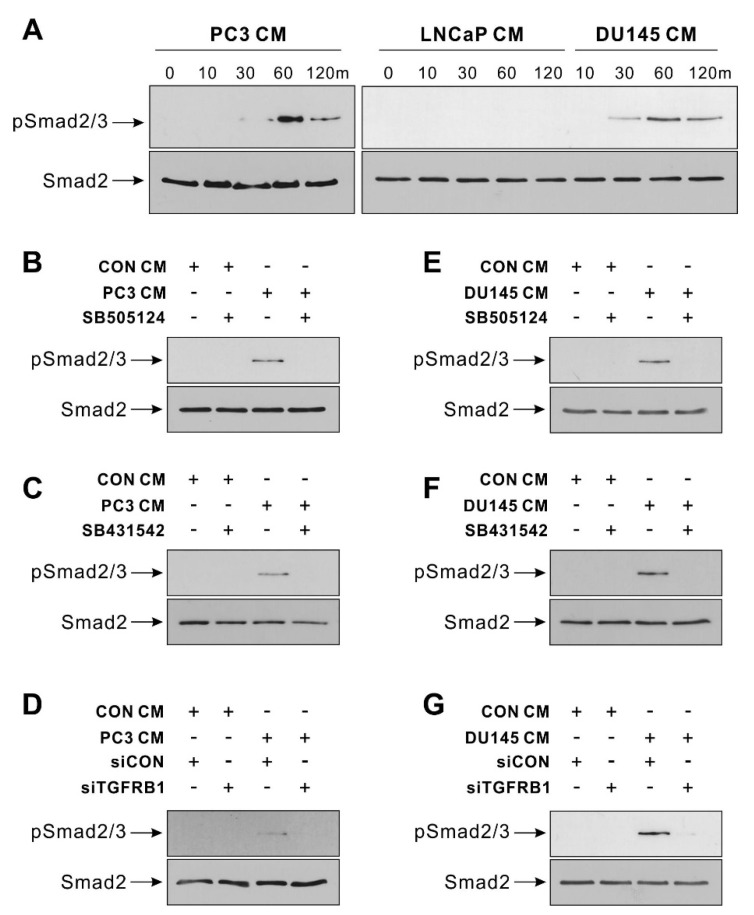
Hormone-insensitive prostate tumor cell-conditioned medium activates TGF-β signaling in bone marrow-derived mesenchymal stem cells (BM-MSCs). (**A**) Western blot analysis of phosphorylated Smad2/3 (p-Smad2/3) and Smad2. BM-MSCs were treated with the control-conditioned medium (CON CM), the PC3-conditioned medium (PC3 CM), the DU145-conditioned medium (DU145 CM), or the LNCaP-conditioned medium (LNCaP CM) for the indicated times. (**B**,**C**) Western blot analysis of p-Smad2/3 and Smad2 in BM-MSCs treated with SB505124 (5 μM) or SB431542 (10 μM) for 30 min prior to treatment with CON CM or PC3 CM for 60 min. (**D**) Western blot analysis of p-Smad2/3 and Smad2. BM-MSCs transfected with control siRNA (siCON) or TGF-β type 1 receptor (siTGFBR1) were treated with CON CM or PC3 CM for 60 min. (**E**,**F**) Western blot analysis of p-Smad2/3 and Smad2 in BM-MSCs treated with SB505124 (5 μM) or SB431542 (10 μM) for 30 min prior to treatment with CON CM or DU145 CM for 60 min. (**G**) Western blot analysis of p-Smad2/3 and Smad2. BM-MSCs transfected with control siRNA (siCON) or TGF-β type 1 receptor (siTGFBR1) were treated with CON CM or DU145 CM for 60 min. Uncropped Western blot images are available in Figure S4.

**Figure 3:**
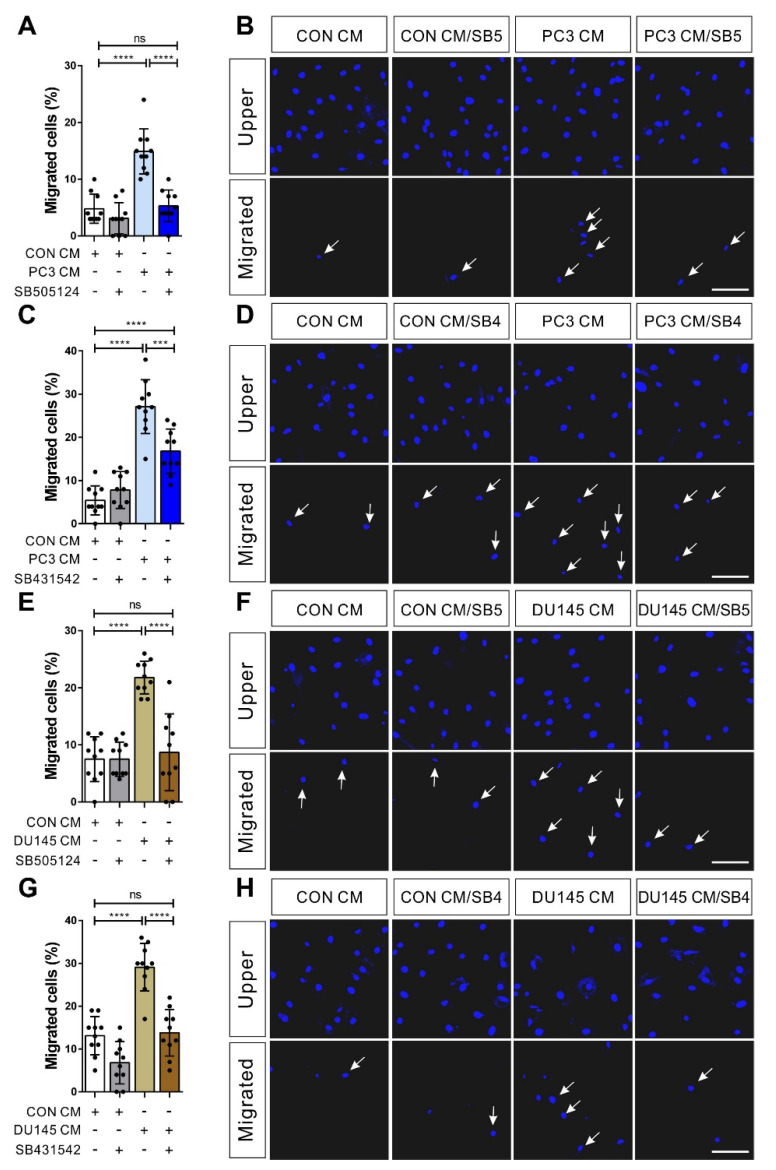
Hormone-insensitive prostate tumor cell-conditioned medium induces the migration of bone marrow-derived mesenchymal stem cells (BM-MSCs) in a TGF-β-dependent manner. (**A**–**D**) Migration of BM-MSCs pretreated with SB505124 (5 μM; (**A**,**B**)) or SB431542 (10 μM; (**C**,**D**)) for 30 min toward the control-conditioned medium (CON CM) or the PC3-conditioned medium (PC3 CM). (**E**–**H**) Migration of BM-MSCs pretreated with SB505124 (5 μM; (**E**,**F**)) or SB431542 (10 μM; (**G**,**H**)) for 30 min toward CON CM or the DU145-conditioned medium (DU145 CM). White arrows indicate DAPI-stained nuclei of migrated BM-MSCs on the lower membrane surface. Results are presented as the mean ± SD (*n* = 2 samples for each group). Scale bars indicate 100 μm. *p*-Value were obtained by *t*-test (*** *p* ≤ 0.001; **** *p* ≤ 0.0001; ns, *p* > 0.05).

The expression level of TGF-β in prostate tumors is positively correlated with higher Gleason scores [22,23]. The expression level of TGF-β1 in prostate cancers was slightly but significantly higher in those with a Gleason score of 8 or higher than in those with a Gleason score of under 8 (TCGA, Figure S2). It is known that the androgen pathway is functionally associated with TGF-β signaling in prostate tumor cells, and the androgen-independent progress of prostate tumors can be predicted by the Gleason score [28,29,39]. Hormone-insensitive prostate tumor cell lines, including PC3 and DU145, significantly express TGF-β1 and TGF-β2 among TGF-β ligands (TGF-β1, TGF-β2, and TGF-β3), but hormone-sensitive prostate tumor cell lines, including LNCaP and VCaP, do not (Cancer Cell Line Encyclopedia; CCLE). PC3 and DU145 expressed the mRNA of TGF-β1, TGF-β2, and TGF-β3, but LNCaP did not (Figure S3). Due to this, BM-MSCs increased the phosphorylation of Smad 2/3 in response to PC3 CM and DU145 CM, but not to LNCaP CM (Figure 2A). The inhibitor of TGF-β type 1 receptors, SB505124, and SB431542 inhibited PC3 CM-induced phosphorylation of Smad2/3 in BM-MSCs (Figure 2B,C). In addition, the siRNA-mediated knockdown of TGF-β type 1 receptor inhibited the phosphorylation of Smad2/3 in BM-MSCs in response to PC3 CM (Figure 2D). The DU145 CM-mediated increase in Smad2/3 phosphorylation of BM-MSCs was downregulated with SB505124, SB431542, or the knockdown of TGF-β type 1 receptor (Figure 2E–G). More importantly, the inhibition of TGF-β signaling also inhibited the migration of BM-MSCs toward the conditioned medium of PC3 and DU145, both of which expressed TGF-β (Figure 3). The inhibitor of TGF-β type 1 receptors, SB505124, and SB431542 inhibited the migration of BM-MSCs toward PC3 CM (Figure 3A–D). The inhibitors also downregulated the migration of BM-MSCs toward DU145 CM (Figure 3E–H).

### 3.3. Hormone-Insensitive Prostate Tumor Cells Enhance the Expression of N-Cadherin in BM-MSCs via TGF-β Signal

**Figure 4:**
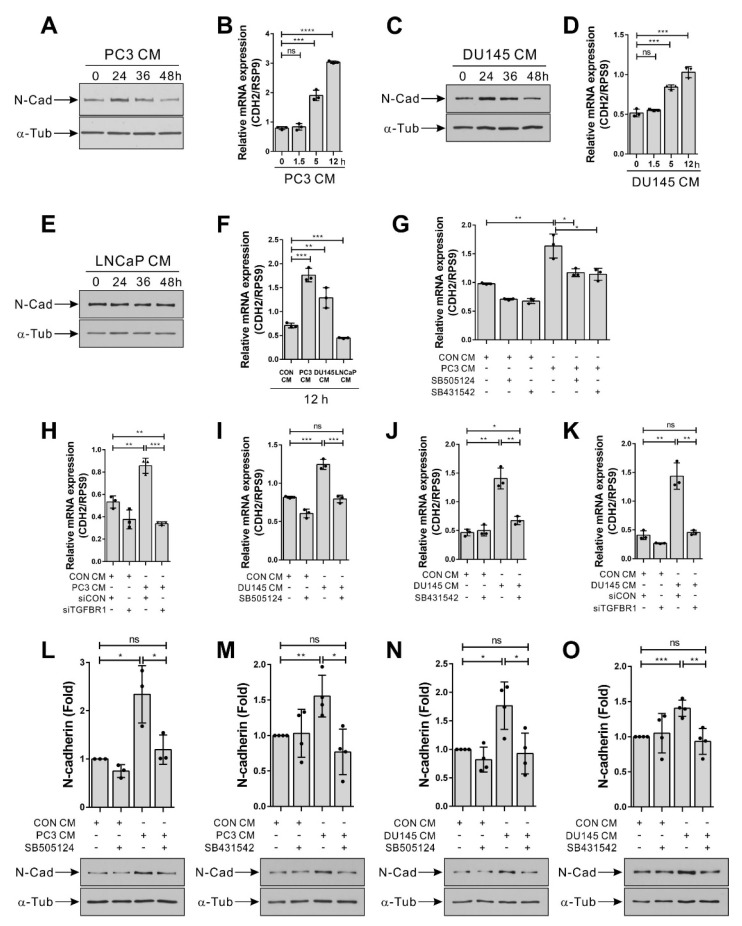
Hormone-insensitive prostate tumor cell-conditioned medium increases the expression of N-cadherin in bone marrow-derived mesenchymal stem cells (BM-MSCs) in a TGF-β-dependent manner. (**A**–**F**) Western blot analysis of N-cadherin (N-cad) and ɑ-tubulin (ɑ-Tub) and qRT-PCR analysis of N-cadherin (CDH2). BM-MSCs were treated with the control-conditioned medium (CON CM), the PC3-conditioned medium (PC3 CM), the DU145-conditioned medium (DU145 CM), or the LNCaP-conditioned medium (LNCaP CM) for the indicated times. (**G**) qRT-PCR analysis of N-cadherin (CDH2) in BM-MSCs pretreated with SB505124 (5 μM) or SB431542 (10 μM) for 30 min prior to treatment with CON CM or PC3 CM for 12 h. (**H**) qRT-PCR analysis of N-cadherin (CDH2) in BM-MSCs transfected with control siRNA (siCON) or TGF-β type 1 receptor siRNA (siTGFBR1) prior to treatment with CON CM or PC3 CM for 12 h. (**I,J**) qRT-PCR analysis of N-cadherin (CDH2) in BM-MSCs pretreated with SB505124 (5 μM) or SB431542 (10 μM) for 30 min prior to treatment with CON CM or DU145 CM for 12 h. (**K**) qRT-PCR analysis of N-cadherin (CDH2) in BM-MSCs transfected with control siRNA (siCON) or TGF-β type 1 receptor siRNA (siTGFBR1) prior to treatment with CON CM or DU145 CM for 12 h. (**L**–**O**) Western blot analysis of N-cadherin (N-cad) and ɑ-tubulin (ɑ-Tub). BM-MSCs were treated with CON CM, PC3 CM, or DU145 CM for 12 h. Results are presented as the mean ± SD (three to four independent experiments). The statistical significance was determined by the *p*-value obtained by t-test (* *p* ≤ 0.05; ** *p* ≤ 0.01; *** *p* ≤ 0.001; **** *p* ≤ 0.0001; ns, *p* > 0.05). Uncropped Western blot images are available in Figure S6.

TGF-β increases the expression of N-cadherin in BM-MSCs, as well as breast tumor cells expressing TGF-β [32,33]. PC3 and DU145 cells, both of which expressed TGF-β, enhanced the N-cadherin expression in BM-MSCs (Figure 4). The expression level of N-cadherin protein increased in BM-MSCs 24 h post-treatment of PC3 CM and gradually decreased to the basal level (Figure 4A). The expression level of N-cadherin mRNA also gradually increased in BM-MSCs in response to PC3 CM (Figure 4B). As PC3 CM increased, DU145 CM also enhanced the expression level of N-cadherin (Figure 4C,D). However, LNCaP CM, which neither expressed TGF-β nor activated TGF-β signaling in BM-MSCs, did not significantly affect the N-cadherin expression level of BM-MSCs (Figure 4E,F). siRNA-mediated knockdown of TGF-β type 1 receptor (Figure S5) prevented a PC3 CM- or DU145 CM-induced increase in the expression level of N-cadherin in BM-MSCs, as well as SB505124 and SB431542 (Figure 4G–K). The PC3 CM- or DU145 CM-induced increase in the expression level of N-cadherin protein was also downregulated with SB505124 and SB431542 (Figure 4L–O).

### 3.4. N-Cadherin Mediates Cell–Cell Adhesion Required for the Collective Migration of BM-MSCs in Response to Hormone-Insensitive Prostate Tumor Cells That Express TGF-β

**Figure 5:**
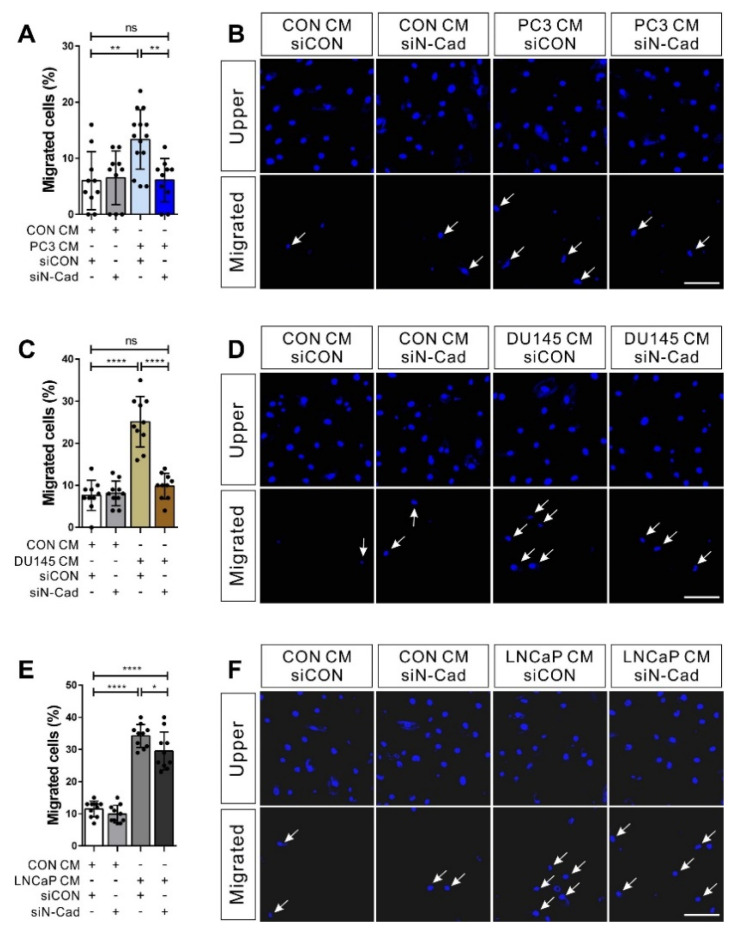
N-cadherin mediates the migration of bone marrow-derived mesenchymal stem cells (BM-MSCs) toward hormone-insensitive prostate tumor cell-conditioned medium. Migration of BM-MSCs transfected with each siRNA in response to prostate tumor cell-conditioned medium. BM-MSCs transfected with control siRNA (siCON) or N-cadherin siRNA (siN-Cad) treated with the PC3-conditioned medium (PC3 CM; (**A**,**B**)), the DU145-conditioned medium (DU145 CM; (**C**,**D**)), the LNCaP-conditioned medium (LNCaP CM; (**E**,**F**)), or the control-conditioned medium (CON CM) for 12 h. White arrows indicate DAPI-stained nuclei of migrated BM-MSCs on the lower membrane surface. Results are presented as the mean ± SD (*n* = 2 samples for each group; two independent experiments). Scale bars indicate 100 μm. The statistical significance was determined by the *p*-value obtained by *t*-test (* *p* ≤ 0.05; ** *p* ≤ 0.01; **** *p* ≤ 0.0001; ns, *p* > 0.05).

N-cadherin is required for the migration of BM-MSCs in response to TGF-β ligand [33] and breast tumor cells expressing TGF-β [32]. Therefore, we investigated whether N-cadherin can mediate the migration of BM-MSCs in response to hormone-insensitive prostate tumor cells expressing TGF-β. siRNA-mediated knockdown of N-cadherin (Figure S7) diminishes the migration of BM-MSCs in response to the conditioned medium of PC3 and DU145, which are hormone-insensitive prostate tumor cells expressing TGF-β (Figure 5A–D). However, the knockdown of N-cadherin very slightly decreased BM-MSCs’ migration in response to the conditioned medium of LNCaP, which is a hormone-sensitive prostate tumor cell not expressing TGF-β (Figure 5E–F).

**Figure 6:**
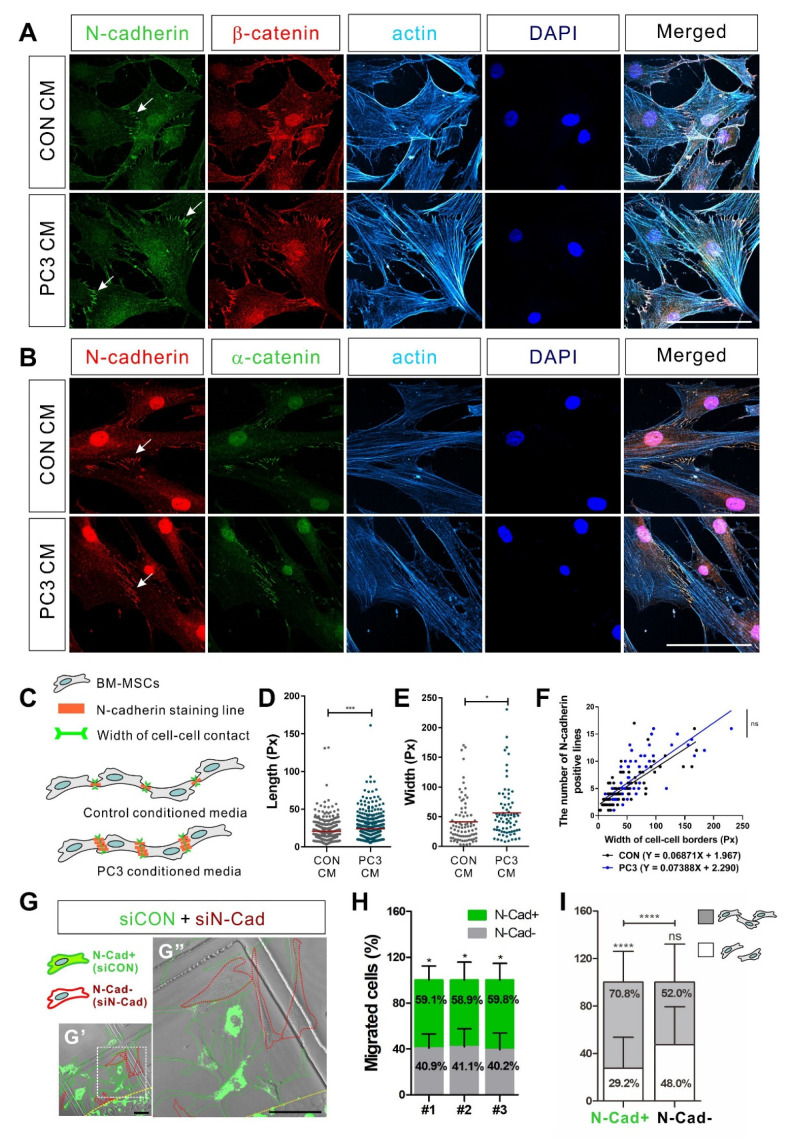
N-cadherin-mediated cell–cell adhesion is required for the migration of bone marrow-derived mesenchymal stem cells (BM-MSCs) toward the PC3-conditioned medium. (**A**,**B**) Immunocytochemistry of N-cadherin, β-catenin, and ɑ-catenin in BM-MSCs treated with the control-conditioned medium (CON CM) or the PC3-conditioned medium (PC3 CM). N-cadherin was stained in green and β-catenin was stained in red (**A**). N-cadherin was stained in red and ɑ-catenin was stained in green (**B**). Actin was stained with phalloidin in sky blue and nuclei were stained with DAPI in blue. White arrows indicate colocalization of N-cadherin and β-catenin or colocalization of N-cadherin and ɑ-catenin at cell–cell adhesion borders. Scale bars indicate 100 μm. (**C**) Schematic representation of measuring the number of N-cadherin positive lines at cell–cell junction-like structures and the width of cell–cell contact composed of N-cadherin positive lines. (**D**) Quantification of the length of N-cadherin positive lines on cell–cell junction-like structures of BM-MSCs treated with CON CM or PC3 CM for 24 h. The red lines indicate the mean values (*n* = 4 samples for each group; Px, pixel). (**E**) Quantification of the width of cell–cell contact composed of N-cadherin positive lines on BM-MSCs treated with CON CM or PC3 CM for 24 h. The red lines indicate the mean values (*n* = 4 samples for each group; Px, pixel). (**F**) Scatter plot showing the correlation between the width of the cell–cell contact borders and the number of N-cadherin positive lines upon cell–cell contact (CON CM in black-filled circles and PC3 CM in blue-filled circles; Px, pixel). Linear regression was applied for correlation analysis. (**G**) Three-dimensional migration using a mixture of BM-MSCs transfected with control siRNA (siCON; stained with calcein AM in green; green-dotted line) or N-cadherin siRNA (siN-Cad; red-dotted line). White dotted boxes in G’ are magnified in G” and yellow broken line indicates the margin of the collagen gel containing cells. Scale bars indicate 100 μm. Percentages of BM-MSCs transfected with siCON or BM-MSCs transfected with siN-Cad among the total number of migrating BM-MSCs from each mixture of cell–collagen gel are shown in (**H**) (*n* = 3 samples). The percentage of migrating cells with or without cell–cell adhesion among the total number of migrating BM-MSCs transfected with siCON (N-Cad+ cells) or the percentage of migrating cells with or without cell–cell adhesion among the total number of migrating BM-MSCs transfected with siN-Cads (N-Cad- cells) are shown in (**I**) (gray bars: Migrated cells with cell–cell adhesion; white bars: Migrated cells without cell–cell adhesion). Results are presented as the mean ± SD (*n* = 3 mixtures of cell–collagen gel). Representative images in Figure S8. *p*-Value obtained by *t*-test or two-way ANOVA (* *p* ≤ 0.05; *** *p* ≤ 0.001; **** *p* ≤ 0.0001; ns, *p* > 0.05).

N-cadherin was co-localized with β-catenin and α-catenin at cell–cell adhesions of BM-MSCs and linked to actin filament in the cytoplasm (Figure 6A,B). Therefore, N-cadherin may consist of adherens junction-like structures at cell–cell adhesions of BM-MSCs. N-cadherin expression upon cell–cell contact of BM-MSCs was investigated by analyzing immunocytochemistry results via two different antibodies against N-cadherin. PC3-conditioned medium (PC3 CM) increased in length of N-cadherin-positive lines upon cell–cell adhesion of BM-MSCs and in width of the cell–cell adhesions (Figure 6A–E). However, there was no change between the number of N-cadherin-positive lines upon cell–cell adhesion of BM-MSCs treated with the conditioned medium of the control (CON CM) or PC3 CM (Figure 6F). Then, we sought the role of N-cadherin in migrating BM-MSCs into hormone-insensitive prostate tumor cells. As N-cadherin mediates the collective migration of BM-MSCs in response to MDA-MB-231, a breast tumor cell line expressing TGF-β [32], N-cadherin may be required for the collective migration of BM-MSCs toward PC3, a hormone-insensitive prostate tumor cell expressing TGF-β. In order to examine this possibility, a 3D cell migration assay was performed using a mixture of BM-MSCs expressing N-cadherin and BM-MSCs, in which N-cadherin was knocked down using transfection of N-cadherin siRNA at a 1:1 ratio. BM-MSCs in which N-cadherin was knocked down migrated less in response to PC3 CM than the control BM-MSCs expressing N-cadherin (Figure 6G,H). Although some populations of BM-MSCs in which N-cadherin was knocked down were still able to migrate in response to PC3 CM, more than 70% of such BM-MSCs showed cell–cell adhesion-free, single-cell migration. In contrast, less than 50% of the migrating cells expressing N-cadherin showed cell–cell adhesion-free migration (Figure 6I). Overall, these results suggest that N-cadherin mediates cell–cell adhesions for the collective migration of BM-MSCs toward hormone-insensitive prostate tumor cells.

## 4. Discussion

Tumor cells interact with resident and recruited non-tumor cells that consist of a tumor microenvironment [40,41,42], as well as with one another. The interaction of tumor cells with non-tumor cells within the tumor microenvironment influences tumor progression and metastasis [43,44]. Bone marrow-derived mesenchymal stem cells (BM-MSCs) are also recruited into the tumor to interact with tumor and non-tumor cells for the formation of the tumor microenvironment [17,18,42,45,46]. Tumor cells secrete various factors, including TGF-β, to recruit non-tumor cells into tumors and to activate the resident and the recruited cells [17,18,42]. In a previous study, we demonstrated that N-cadherin mediates the cell–cell adhesion-dependent collective migration of BM-MSCs in vitro in response to breast tumor cells expressing TGF-β [32]. However, it remains unclear which specific intrinsic molecules mediating cell motility contribute to the recruitment of BM-MSCs into prostate tumor. A key feature of prostate tumor progress is a transition from a hormone-sensitive, androgen-dependent stage to a hormone-insensitive, androgen-independent tumor [3,4,5,6,21,47], and BM-MSCs recruited into prostate tumor contribute to this transition [3]. The expression level of TGF-β in prostate tumors is positively associated with the aggressive features of prostate tumors such as bone metastasis and the Gleason score [22,23]. Bone metastasis develops in around 30% patients with hormone-insensitive stage prostate tumor [48], and the induction of genes involved in metastasis and epithelial-–mesenchymal transition are associated with the transition from the hormone-sensitive stage of prostate tumor to hormone-insensitive stage [49]. In the present study, we demonstrated that N-cadherin mediates the migration of BM-MSCs toward conditions mimicking hormone-insensitive prostate tumor cells expressing TGF-β. However, BM-MSCs migrated in response to hormone-sensitive prostate tumor cells expressing no TGF-β independently on N-cadherin.

Cells have been shown to migrate as individual cells or in groups where cells collectively emerge in a coordinated manner [50]. The collective migration mediated via physical and mechanical interaction between cells is necessary for embryonic morphogenesis, injury regeneration, and tumor metastasis [50,51]. Mesenchymal cells, such as neural crest cells and BM-MSCs, can migrate in a collective manner dependently on cadherins, transmembrane proteins that mediate cell–cell interaction, in response to various stimuli [32,52,53]. N-cadherin-dependent cell–cell adhesion induces directional information in a group of cells via polarization of Rac1 activity in response to SDF-1 [53]. N-cadherin in cell–cell adhesion between BM-MSCs was required for cell–cell contact-dependent migration of BM-MSCs toward the hormone-insensitive prostate tumor cells expressing TGF-β, as N-cadherin mediated the collective migration of BM-MSCs toward breast tumor cells expressing TGF-β [32]. Importantly, BM-MSCs were also able to collectively migrate toward hormone-sensitive prostate tumor cells that do not express TGF-β, but N-cadherin was not required for the migration of BM-MSCs. Adhesion molecules other than N-cadherin, such as junctional adhesion molecules (JAMs) and cell adhesion molecules (CAMs), may contribute to TGF-β-independent collective migration of BM-MSCs toward hormone-sensitive prostate tumor cells. Further experiments are required to investigate the molecules. Around 70% of the total migrating BM-MSCs toward the conditioned medium of PC3 showed the characteristics of collective migration. Although N-cadherin knockdown decreased the number of the collectively migrating BM-MSCs, a significant number of migrating BM-MSCs maintained cell–cell contact. Therefore, other molecules that contribute to the formation of cell–cell adhesion of BM-MSCs may also mediate the collective migration of BM-MSCs independently on N-cadherin. Mechanisms by which N-cadherin-mediated cell–cell interactions between BM-MSCs mediate the collective migration of BM-MSCs toward prostate tumor cells expressing TGF-β and molecules that contribute to BM-MSC interaction for collective migration toward prostate tumor cells remain unclear. Further experiments are required to investigate such mechanisms and molecules.

The expression of N-cadherin upon cell–cell adhesion of BM-MSCs was enhanced by TGF-β expressed by hormone-insensitive prostate tumor cells, not by hormone-sensitive prostate tumor cells not expressing TGF-β. It has been demonstrated that TGF-β increases the N-cadherin expression in BM-MSCs dependently on Smad4 [32]. Smad4 knockdown mediated by siRNA did not block the increase in N-cadherin of BM-MSCs in response to hormone-insensitive prostate tumor cells (unpublished), although the increase in N-cadherin expression was significantly downregulated in response to hormone-insensitive prostate tumor cells by inhibiting TGF-β with either TGF-β type I receptor inhibitors or TGF-β type I receptor knockdown. These results suggest that N-cadherin may be non-canonically induced in a Smad4-independent manner by TGF-β expressed by hormone-insensitive prostate tumor cells. Further experiments are required to examine this possibility.

The present study suggests that BM-MSCs migrated toward either hormone-insensitive or -sensitive prostate tumor cells in response to different stimuli via different intrinsic molecules that mediated their migration. It is unclear whether BM-MSCs recruited into hormone-insensitive or -sensitive tumors function differently and contribute to shaping the functionally different microenvironments of tumors. BM-MSCs recruited into hormone-sensitive prostate tumors might lead to the formation of a tumor microenvironment that induces hormone-insensitivity in tumor cells. Meanwhile, BM-MSCs recruited into hormone-insensitive prostate tumors might form a tumor microenvironment that evolves a more aggressive phenotype in tumor cells via various mechanisms, such as recruitment of and polarization into M2-type macrophages [18]. Further experiments are necessary to investigate these possibilities.

## 5. Conclusions

The current study provides evidence that N-cadherin composing adherens junction-like structures upon cell–cell contact of BM-MSCs mediates the migration of BM-MSCs toward hormone-insensitive prostate tumors in a TGF-β signaling-dependent manner. Our results suggest a promising novel strategy for re-engineering the prostate tumor microenvironment. N-cadherin can be targeted to prevent BM-MSCs from being recruited into hormone-insensitive prostate tumors and contributing to the formation of a tumor microenvironment with a high metastasis risk.

## Data Availability

The datasets generated during and/or analyzed during the current study are available from the corresponding author on reasonable request.

## Supplementary Materials

The following are available online at https://www.mdpi.com/article/10.3390/biomedicines9111572/s1, Figure S1: The measurement of the number and length of N-cadherin-positive structures at the borders between BM-MSCs and the width of N-cadherin-positive borders between BM-MSCs; Figure S2: Expression of the TGF-β1 ligand in prostate cancer; Figure S3: Expression of TGF-β ligands (TGF-β1, TGF-β2, and TGF-β3) in PC3, DU145, and LNCaP prostate tumor cells; Figure S4: Full unedited blots of Figure 2; Figure S5: Verification of siRNA-mediated knockdown of TGF-β type 1 receptor; Figure S6: Full unedited blots of Figure 4; Figure S7: Verification of siRNA-mediated knockdown of N-cadherin; Figure S8. Representative images used for the analysis of Figure 6H,I.
