# Supplementary material for: Bone Marrow-Derived Mesenchymal Stem Cells Migrate toward Hormone-Insensitive Prostate Tumor Cells Expressing TGF-β via N-Cadherin
Source: Biomedicines. 2021 Oct 29;9(11):1572. doi: 10.3390/biomedicines9111572 (PMC8615076; doi:10.3390/biomedicines9111572)
Supplement: Supplementary file 1 [file biomedicines-09-01572-s001.zip › Supplementary Materials/biomedicines-1419116_Supplementary_Figure_updated.pdf]

Bone marrow-derived mesenchymal stem cells migrate toward hormone-insensitive prostate tumor cells expressing TGF-β via N-cadherin

Jinok Noh [1], Jinyeong Yu [2], Wootak Kim [1], Aran Park [2] and Ki-Sook Park [1,3,*]

1 Department of Biomedical Science and Technology, Graduate School, Kyung Hee University, Seoul 02447, Korea; rjo8853@khu.ac.kr (J.N.); kimwotak@khu.ac.kr (W.K.)
2 Graduate School of Biotechnology, Kyung Hee University, Yongin 17104, Korea; jinyeong90@khu.ac.kr (J.Y.); arvi2114@khu.ac.kr (A.P.)
3 East-West Medical Research Institute, Kyung Hee University, Seoul 02447, Korea; kisookpark@khu.ac.kr (K.-S.P.)
* Correspondence: kisookpark@khu.ac.kr; Tel.: +82-2-958-9368 (K.-S.P.)

Supplementary Figure S1. The measurement of the number and the length of N-cadherin-positive structures at the borders between BM-MSCs and the width of N-cadherin-positive borders between BM-MSCs.
Supplementary Figure S2. The expression of TGF-β1 ligand in prostate cancers.
Supplementary Figure S3. Expression of TGF-β ligands (TGF-β1, TGF-β2, and TGF-β3) in PC3, DU145, and LNCaP prostate tumor cells.
Supplementary Figure S4: Full unedited blots of Figure 2.
Supplementary Figure S5. Verification of siRNA-mediated knockdown of TGF-β type 1 receptor.
Supplementary Figure S6: Full unedited blots of Figure 4.
Supplementary Figure S7. Verification of siRNA-mediated knockdown of N-cadherin.
Supplementary Figure S8. Representative images used for analysis of Figure 6H and I.

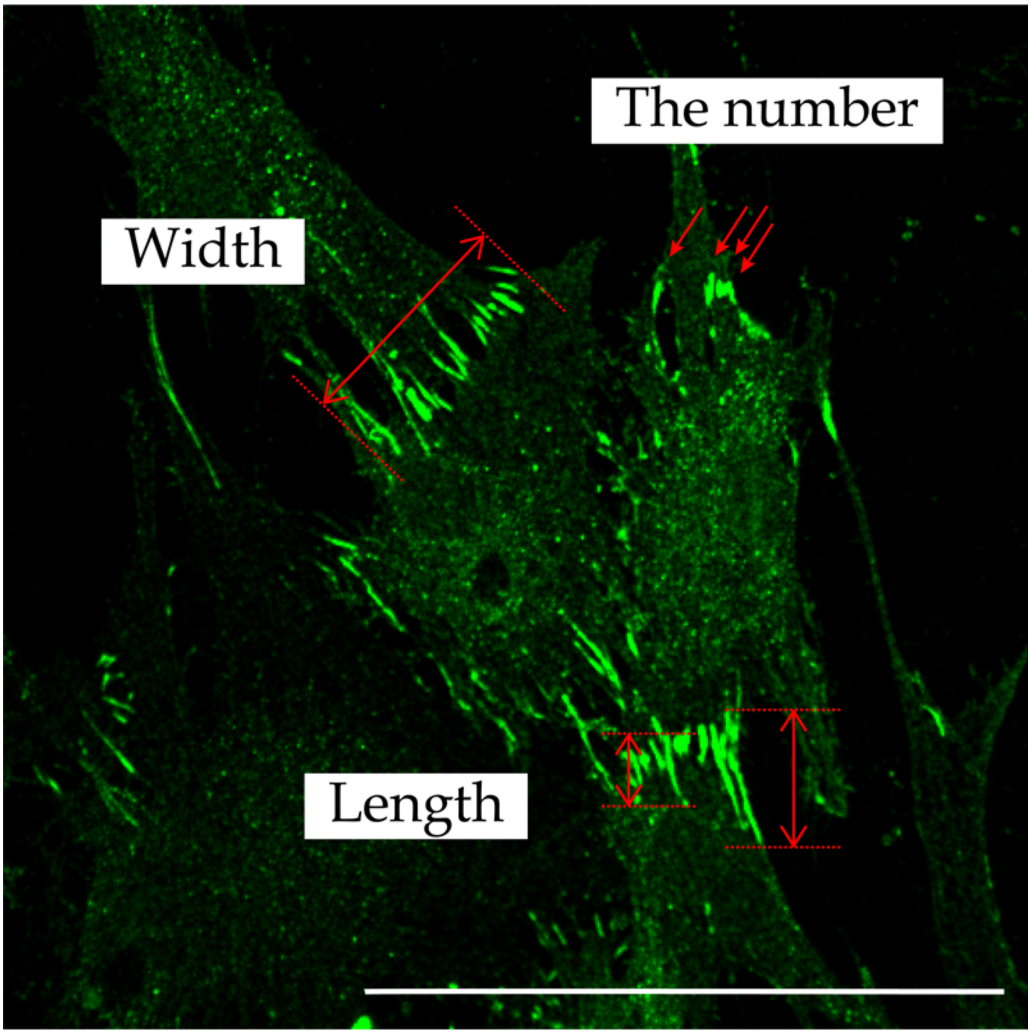

Supplementary Figure S1. The measurement of the number and the length of N-cadherin-positive structures at the borders between BM-MSCs and the width of N-cadherin-positive borders between BM-MSCs. N-cadherin was stained in green and scale bar indicates 100 μm.

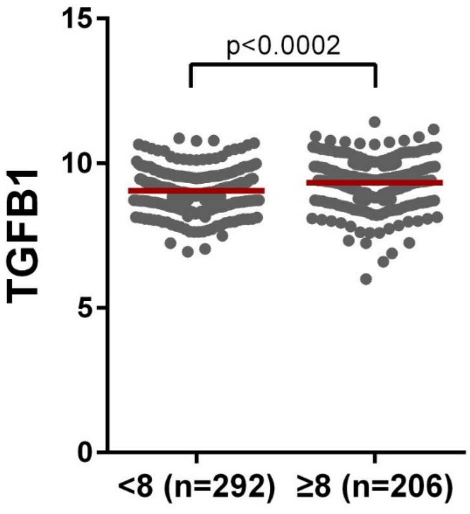

Supplementary Figure S2. The expression of TGF-β1 ligand in prostate cancers. The RNA-seq database of human prostate cancers from The Cancer Genome Atlas (TCGA) was analyzed. There were 292 samples with a Gleason score of under 8 and 206 with a Gleason score of higher or equal to 8. The red lines indicate the mean values.

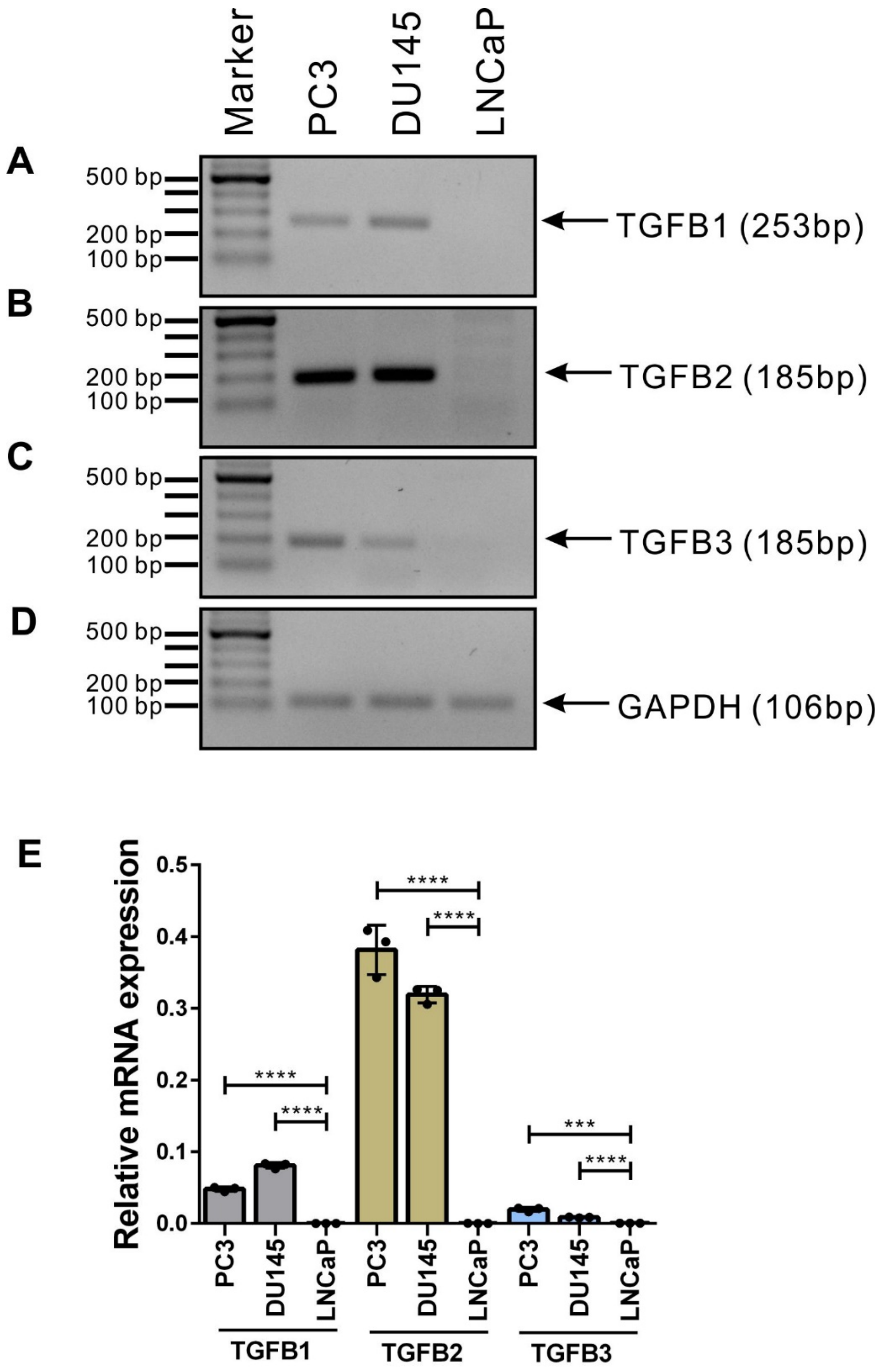

Supplementary Figure S3. Expression of TGF-β ligands (TGF-β1, TGF-β2, and TGF-β3) in PC3, DU145, and LNCaP prostate tumor cells. Semi-quantitative RT-PCR analysis of TGF-β1 (TGFB1, **A**), TGF-β2 (TGFB2, **B**), and TGF-β3 (TGFB3, **C**) in PC3, DU145, and LNCaP. Glyceraldehyde 3-phosphate dehydrogenase gene (GAPDH, **D**) was used as an endogenous control for semi-quantitative RT-PCR analysis. Quantitative RT-PCR (qRT-PCR) analysis of TGFB1, TGFB2, and TGFB3 in PC3, DU145, and LNCaP (**E**). p Value obtained by t-test (***, $p \leq 0.001$; ****, $p \leq 0.0001$). Ribosomal protein S9 gene (RPS9) was used as an endogenous control for qRT-PCR analysis.

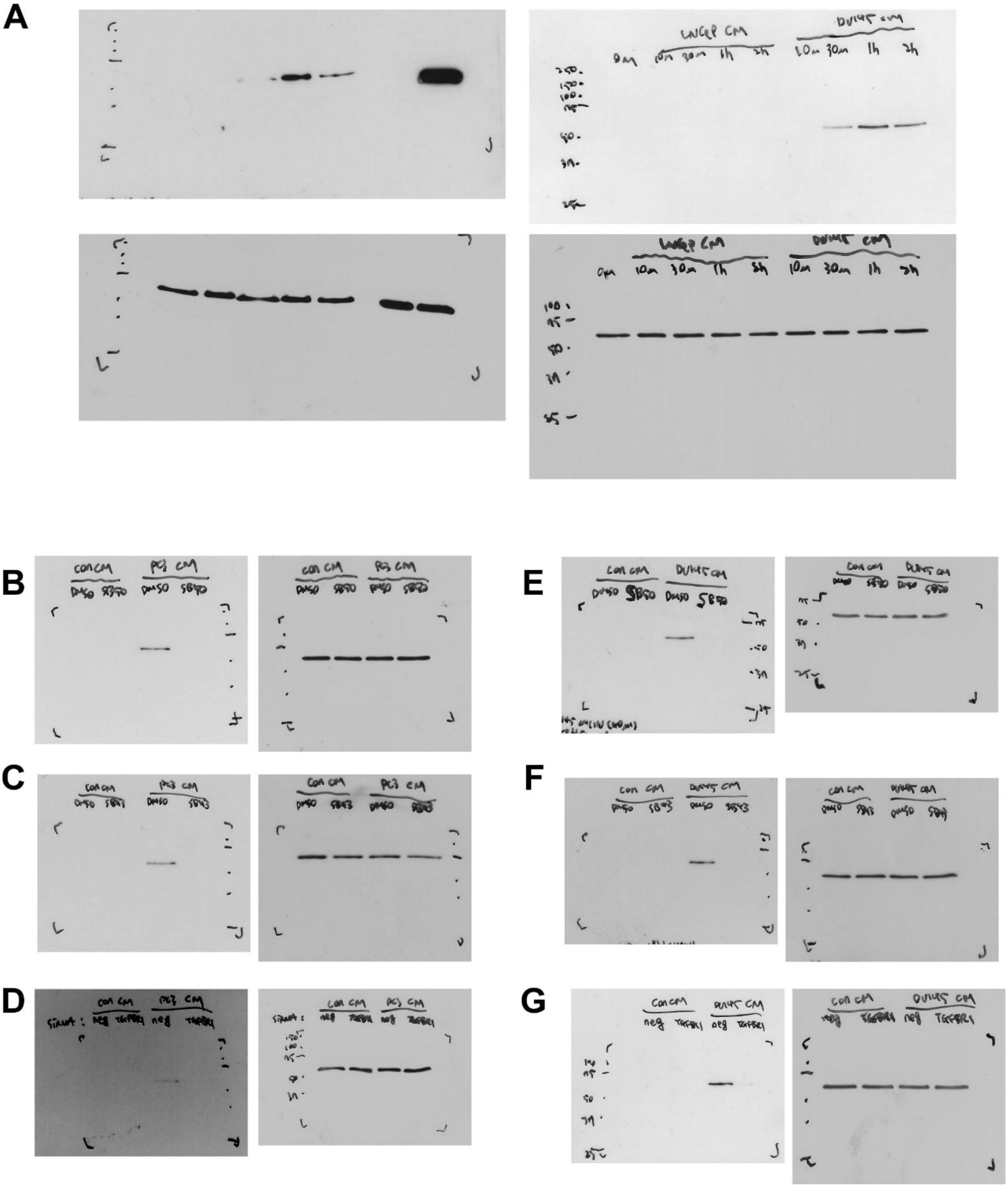

Supplementary Figure S4: Full unedited blots of Figure 2. (**A**) Full unedited blots for pSmad2/3 and Smad2 of Figure 2A. (**B**) Full unedited blots for pSmad2/3 and Smad2 of Figure 2B. (**C**) Full unedited blots for pSmad2/3 and Smad2 of Figure 2C. (**D**) Full unedited blots for pSmad2/3 and Smad2 of Figure 2D. (**E**) Full unedited blots for pSmad2/3 and Smad2 of Figure 2E. (**F**) Full unedited blots for pSmad2/3 and Smad2 of Figure 2F. (**G**) Full unedited blots for pSmad2/3 and Smad2 of Figure 2G.

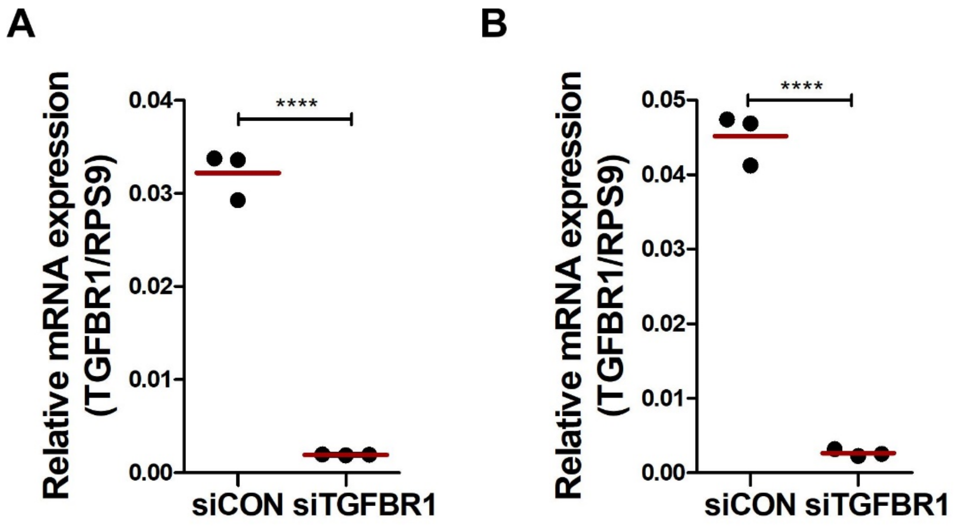

Supplementary Figure S5. Verification of siRNA-mediated knockdown of TGF-β type 1 receptor. qRT-PCR analysis of TGF-β type 1 receptor (TGFBR1). BM-MSCs were transfected with control siRNA (siCON) or TGF-β type 1 receptor siRNA (siTGFBR1) prior to treatment with PC3 conditioned medium **(A)** or DU145 conditioned medium **(B)**. Ribosomal protein S9 gene (RPS9) was used as an endogenous control.

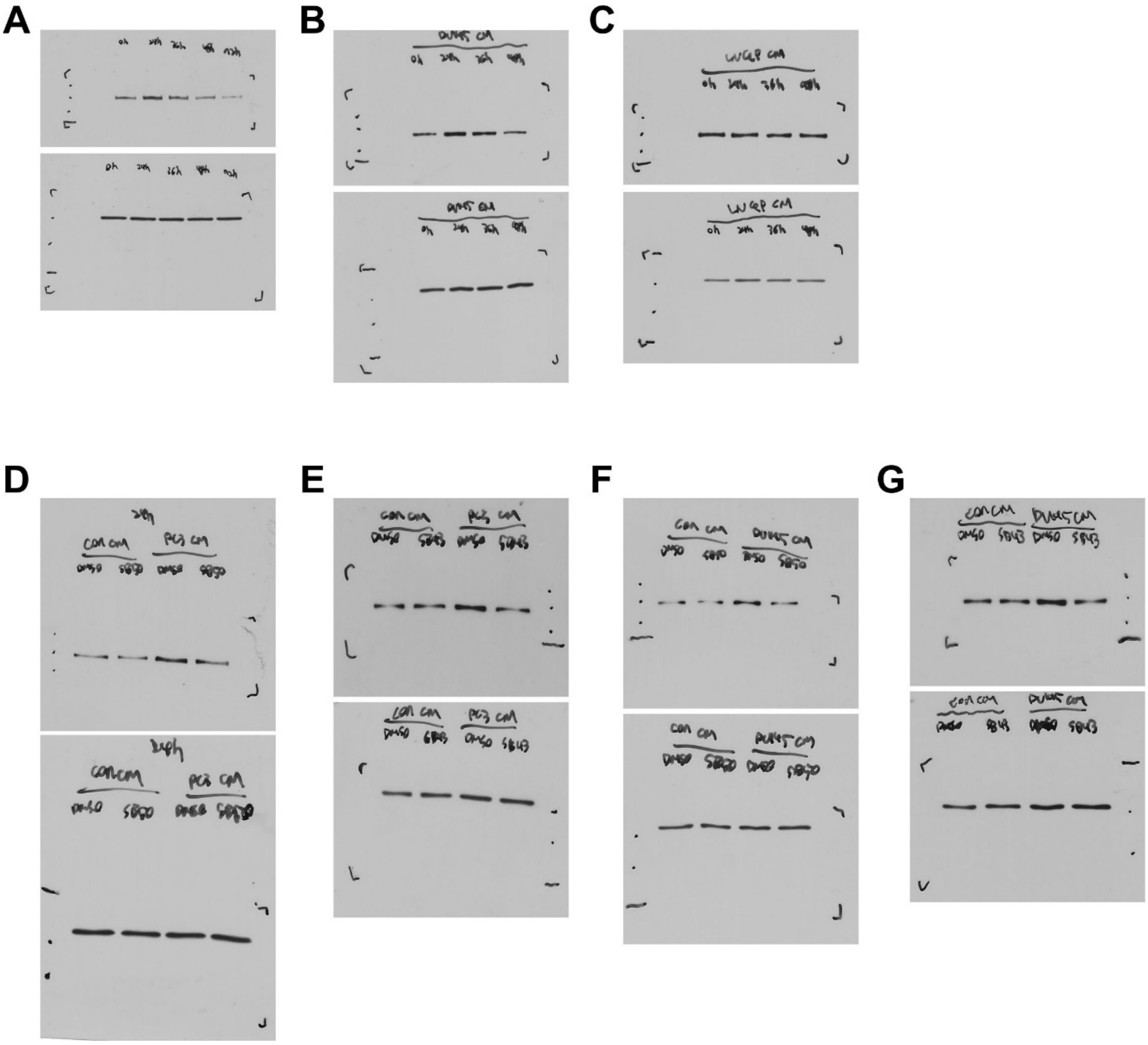

Supplementary Figure S6: Full unedited blots of Figure 4. (**A**) Full unedited blots for N-cadherin (N-cad) and α-tubulin (α-Tub) of Figure 4A. (**B**) Full unedited blots for N-cadherin (N-cad) and α-tubulin (α-Tub) of Figure 4C. (**C**) Full unedited blots for N-cadherin (N-cad) and α-tubulin (α-Tub) of Figure 4E. (**D**) Full unedited blots for N-cadherin (N-cad) and α-tubulin (α-Tub) of Figure 4L. (**E**) Full unedited blots for N-cadherin (N-cad) and α-tubulin (α-Tub) of Figure 4M. (**F**) Full unedited blots for N-cadherin (N-cad) and α-tubulin (α-Tub) of Figure 4N. (**G**) Full unedited blots for N-cadherin (N-cad) and α-tubulin (α-Tub) of Figure 4O.

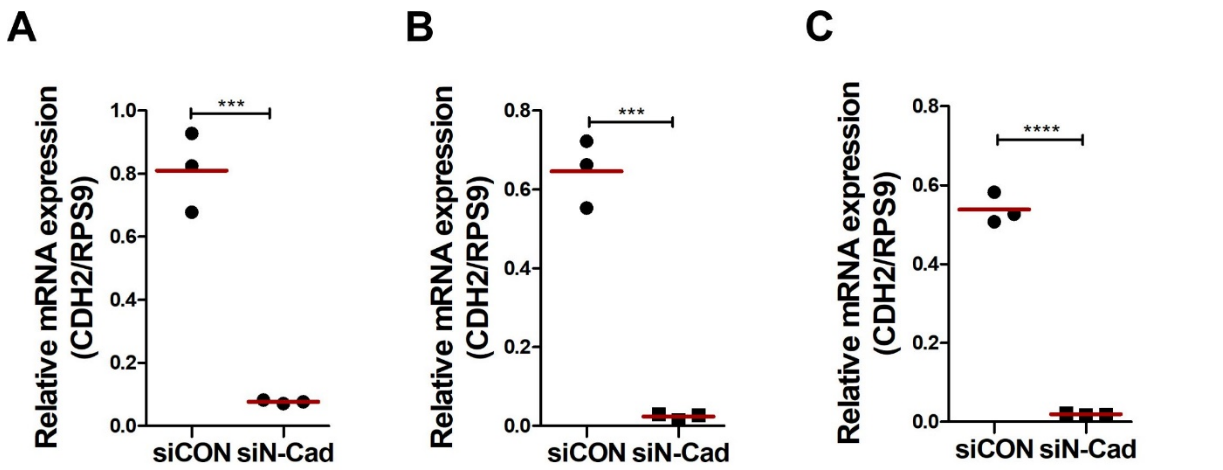

Supplementary Figure S7. Verification of siRNA-mediated knockdown of N-cadherin. qRT-PCR analysis of N-cadherin (CDH2). BM-MSCs were transfected with control siRNA (siCON) or N-cadherin siRNA (siN-Cad) prior to evaluating BM-MSCs migration in response to PC3 conditioned medium **(A),** DU145 conditioned medium **(B)** or LNCaP conditioned medium **(C)**. Ribosomal protein S9 gene (RPS9) was used as an endogenous control.

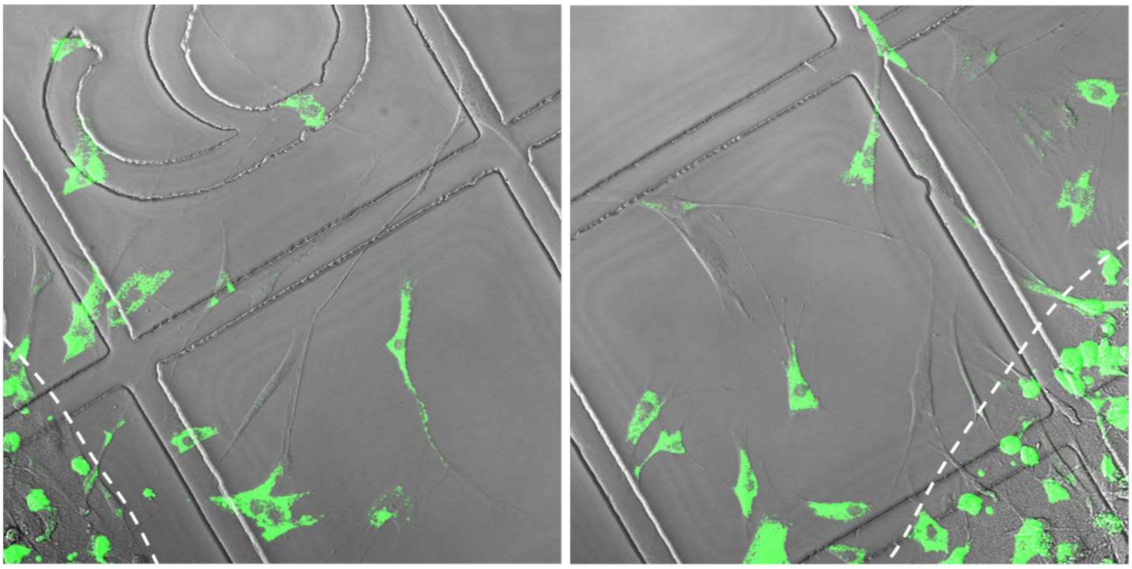

N-cad+	N-cad KD	total	N-cad+ migrated cells(%)	N-cad KD migrated cells(%)
8	3	11	73%	27%

N-cad+	N-cad KD	total	N-cad+ migrated cells(%)	N-cad KD migrated cells(%)
12	7	19	63%	37%

Supplementary Figure S8. Representative images used for analysis of Figure 6H and I. White broken lines indicate the margin of the collagen gel containing cells.
